# Toxicogenomic analysis incorporating operon-transcriptional coupling and toxicant concentration-expression response: analysis of MX-treated *Salmonella*

**DOI:** 10.1186/1471-2105-8-378

**Published:** 2007-10-09

**Authors:** William O Ward, Carol D Swartz, Steffen Porwollik, Sarah H Warren, Nancy M Hanley, Geremy W Knapp, Michael McClelland, David M DeMarini

**Affiliations:** 1 Environmental Carcinogenesis Division, US Environmental Protection Agency, Research Triangle Park, NC 27711, USA; 2 Department of Environmental Science & Engineering, University of North Carolina, Chapel Hill, NC 27599, USA; 3 Sidney Kimmel Cancer Center, San Diego, CA 92121, USA

## Abstract

**Background:**

Deficiencies in microarray technology cause unwanted variation in the hybridization signal, obscuring the true measurements of intracellular transcript levels. Here we describe a general method that can improve microarray analysis of toxicant-exposed cells that uses the intrinsic power of transcriptional coupling and toxicant concentration-expression response data. To illustrate this approach, we characterized changes in global gene expression induced in *Salmonella typhimurium *TA100 by 3-chloro-4-(dichloromethyl)-5-hydroxy-2(*5H*)-furanone (MX), the primary mutagen in chlorinated drinking water. We used the co-expression of genes within an operon and the monotonic increases or decreases in gene expression relative to increasing toxicant concentration to augment our identification of differentially expressed genes beyond Bayesian-t analysis.

**Results:**

Operon analysis increased the number of altered genes by 95% from the list identified by a Bayesian t-test of control to the highest concentration of MX. Monotonic analysis added 46% more genes. A functional analysis of the resulting 448 differentially expressed genes yielded functional changes beyond what would be expected from only the mutagenic properties of MX. In addition to gene-expression changes in DNA-damage response, MX induced changes in expression of genes involved in membrane transport and porphyrin metabolism, among other biological processes. The disruption of porphyrin metabolism might be attributable to the structural similarity of MX, which is a chlorinated furanone, to ligands indigenous to the porphyrin metabolism pathway. Interestingly, our results indicate that the *lexA *regulon in *Salmonella*, which partially mediates the response to DNA damage, may contain only 60% of the genes present in this regulon in *E. coli*. In addition, *nanH *was found to be highly induced by MX and contains a putative *lexA *regulatory motif in its regulatory region, suggesting that it may be regulated by *lexA*.

**Conclusion:**

Operon and monotonic analyses improved the determination of differentially expressed genes beyond that of Bayesian-t analysis, showing that MX alters cellular metabolism involving pathways other than DNA damage. Because co-expression of similarly functioning genes also occurs in eukaryotes, this method has general applicability for improving analysis of toxicogenomic data.

## Background

Deficiencies in microarray technology can produce undesired variation in the hybridization signal, obscuring a clear measurement of intracellular transcript levels. In order to overcome this problem, we applied two analytical techniques in addition to the typically used t-test to discern differentially expressed genes. We used (a) an operon analysis that assumes if one gene in an operon is differentially expressed, then all genes in that operon are differentially expressed and (b) an analysis relating monotonic-expression response to increasing concentrations of MX.

Many bacterial genes are grouped into multi-gene transcriptional units or operons, resulting in coordinate transcriptional regulation. This has been exploited previously to evaluate different statistical tools available for microarray analyses [1,2]. Other investigators have estimated expression levels by borrowing information from genes within the same operon [3] or have estimated systematic error to increase confidence in significance calls [4]. These studies focused on improving significance calls for individual genes. Our analysis differs from these by identifying changes to functional pathways due to co-expression of genes within an operon. Although previous studies have used monotonic increases in toxicant concentration-gene expression response to identify genes affected by toxicant exposure [5], we have combined this analysis with the operon analysis to construct a list of differentially expressed genes. The resulting list of genes was then analyzed for functional and KEGG pathway representation.

To examine the usefulness of this approach, we have evaluated global gene expression in *Salmonella typhimurium *TA100 by 3-chloro-4-(dichloromethyl)-5-hydroxy-2(*5H*)-furanone (MX), the primary mutagen in chlorinated drinking water. MX is a chlorinated furanone that accounts for 20-60% of the mutagenic activity of chlorinated drinking water and is a multi-site carcinogen in rats [6]. MX and its structural analogues have been given a high or high-moderate rating for priority concern regarding their potential carcinogenicity in drinking water [7]. Although MX is not a regulated drinking water disinfection by-product, it has a relatively high mean cancer potency estimate of 2.3 (mg/kg-d)^-1 ^[6]. It is a potent direct-acting mutagen in *Salmonella *that induces primarily GC to TA base substitutions in bacteria and mammalian cells, presumably as a consequence of replication past un-repaired abasic sites resulting from unstable MX adducts on guanine [6].

Most studies of global gene expression in cells exposed to a toxicant in vitro have measured cytotoxicity as the relevant biological endpoint and were conducted at only one concentration of the toxicant [8]. In addition to cytotoxicity, global gene expression could be determined under conditions of mutagenesis, which generally require relatively high survival to permit viable mutants to be grown under selective conditions. Also, mutagenicity experiments are generally performed under a range of concentrations of the mutagen in order to generate a mutagenicity dose-response curve. Characterization of global gene expression in cells exposed in vitro under conditions of mutagenesis could help reveal the pleiotropic effects of mutagens.

To our knowledge, only a few studies have determined global gene expression in cells exposed in vitro under conditions of mutagenesis [9-12], and all of these were performed in mammalian cells in vitro. Along with changes in expression of DNA repair and metabolism genes, these studies identified other pathways affected by the mutagens tested. Other studies have shown that mutagens alter gene expression in a variety of pathways beyond those involved in mutagenesis per se [13-15]. However, to date, no such study has been performed in the *Salmonella *(Ames) mutagenicity assay, which is the assay used most widely in genetic toxicology. Although Porwollik et al. [16] did evaluate global gene expression in *Salmonella *LT2, which is the parent strain of the Ames strains, they did not report survival or perform mutagenesis after hydrogen peroxide treatment of the cells. They found that hydrogen peroxide induced expression of *sulA*, which is a gene involved in the inhibition of cell division. This somewhat parallels the finding described above in mouse lymphoma cells [9] in which hydrogen peroxide induced genes involved in apoptosis. Consistent with this was the finding in *Salmonella *that hydrogen peroxide depressed expression of genes involved in cell replication and protein synthesis [16]. Thus, in addition to showing the value of the bioinformatic analyses described here, our study also extends the limited literature on the changes in global gene expression in cells treated under conditions of mutagenesis.

## Results

### Mutagenicity

**Figure 1:**
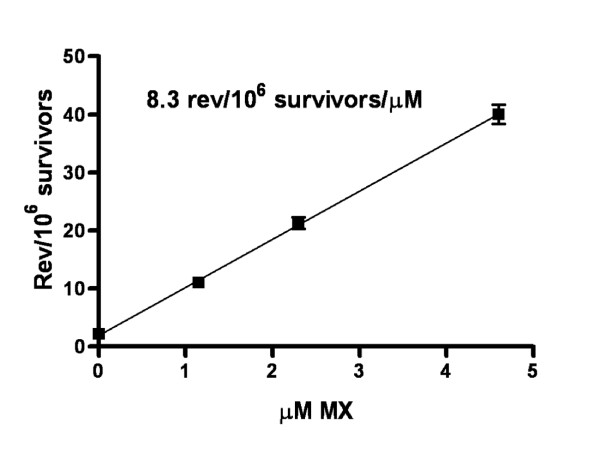
Average mutagenicity of MX in *Salmonella *TA100 from four experiments. The average % survival of log-phase cells was 95, 97, and 73% at 1.15-, 2.3-, and 4.6-μM MX, respectively.

The mutagenic potency of MX was 8.3 revertants/10^6 ^survivors/μM, and MX produced a linear concentration-response curve (Fig. 1) with mutant frequency (revertants/10^6 ^survivors) and survival values at the following concentrations: 0 μM (2.2, 100%), 1.15 μM (11.1, 95%), 2.3 μM (21.3, 97%), and 4.6 μM (40.0, 73%). Thus, MX induced an 18-fold increase in mutant frequency at the highest concentration.

### Principal component analyses (PCA)

**Figure 2:**
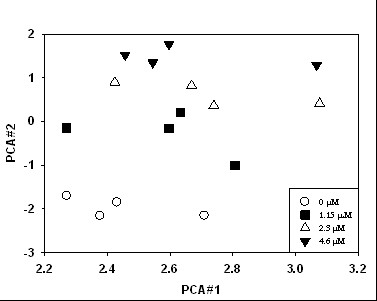
Principal component analysis of differentially expressed genes in MX-treated *Salmonella *TA100. PCA#1 accounts for 31% of the variation in the data and PCA#2 accounts for 16%.

Microarray data were first analyzed as described previously [16]. CyberT, which is an unpaired Bayesian t-test [17], was used to identify those genes whose expression was significantly different (*p *< 0.05) between the control and the highest MX concentration. To determine similarity of expression for biological replicates, those genes were then analyzed by Cluster 3.0 [18] for principal components at all concentrations. In our case, samples have similar PCA #1 values. This is often found in genomic studies and is attributed to biological variation. PCA #2 segregated each concentration of MX, and biological replicates had similar PCA#2 values (Fig. 2). Thus, gene expression changed coordinately with increasing concentrations of MX.

### Operon analysis

The RegulonDB [19] identifies operons and their gene constituents in *E. coli*. To map the *Salmonella *genes on our microarray to *E. coli *genes in the RegulonDB, one author (M. McClelland) blasted a sliding window of 100 bp from the arrayed sequences to the *E. coli *genome and recorded the highest or "best" percentage hit for each arrayed sequence. The median of all "best hits" was approximately 85%. Using the RegulonDB [19] and this ortholog map, all of the genes were assigned to their respective operons. All the genes in an operon were considered differentially expressed when at least one gene in the operon was determined to be differentially expressed at the highest concentration of MX by CyberT analysis (*p *< 0.05). There were 54 operons with at least one differentially expressed gene.

To validate this approach, we evaluated the distribution of *p*-values for all the genes in these 54 operons (Table 1) and found that the *p*-values for these genes were shifted significantly to lower values (0–0.2) as determined by a hyper-geometric test (*p*-value of 1.7 × 10^-11^). To further support this determination of gene co-expression within an operon, we analyzed, in detail the distribution of *p*-values and the uniformity of fold change within these operons (Tables 2 and 3). Fifty-one percent (87/169) of the genes with *p*-values <0.05 clustered in our identified 54 operons or 6% (54/866) of the total number of operons. Ninety-six percent of the 54 operons had genes whose expression moved in one direction.

**Table 1:** Distribution of genes among a range of *p*-values identified by various analyses

	Percent (%) of genes distributed across the range of *p*-values			
	
Range of *p*-values	All genes in the genome*	All genes in selected operons^†^	Non-significant genes in selected operons^‡^	Genes identified by monotonic analysis^§^
0.0 – 0.2	15	72	37	94
0.2 – 0.4	17	11	24	6
0.4 – 0.6	20	6	14	0
0.6 – 0.8	23	5	12	0
0.8 – 1.0	25	6	13	0

*p-values were calculated by CyberT comparisons of gene expression between the control cells and those treated with the highest concentration of MX (4.6 μM) for all 4253 genes analyzed. Thus, 4253 was the denominator used to calculate the percentages in this column. CyberT analysis identified 169 genes having *p*-values < 0.05.

^†^Selected operons were those that had at least one gene with a consistent CyberT-calculated *p*-value < 0.05. Of the 169 genes identified by CyberT analysis, 86 resided in a total of 54 multi-gene operons, and the remaining 83 genes were in single-gene operons. Adding the additional genes on the 54 multi-gene operons (143) to those identified solely by CyberT (169) brought the total genes considered for analysis by the operon method to 312, which was the denominator used to calculate the percentages in this column. A Monte Carlo simulation, repeated 1500 times using a sample size of 312, randomly selected from 4253 *p*-values, and sorted into the *p*-value bins shown in column 1, produced a *p*-value distribution the same to the nearest percentage point as that shown in the second column, i.e., a distribution essentially identical to that found for all genes in the genome.

^‡^There were 161 genes residing in the selected operons that did not have *p*-values < 0.05. Eleven percent of these genes had missing data and, thus, did not appear in the table. Thus, 143 was the denominator used to calculate the percentages in this column. A Monte Carlo simulation repeated 1500 times using a sample size of 143 produced a *p*-value distribution the same to the nearest percentage point as that shown in the second column, i.e., a distribution essentially identical to that found for all genes in the genome.

^§^The monotonic analysis alone identified 309 genes as being differentially expressed. Of these, 153 were uniquely identified by the monotonic analysis; the other 156 had been identified by either the CyberT and/or operon analyses. Thus, the monotonic analysis added an additional 153 genes for analysis, and this number was the denominator used to calculate the percentages shown in this column.

**Table 2:** Analysis of genes considered differentially expressed on the 54 operons identified by the operon analysis

Operon number*	Number of genes in operon	Smallest *p*-value^†^	FC^‡^	Largest *p*-value^§^	FC^¶^	% In same direction^¥^	% with *p *< 0.05	% Missing genes^#^
1123^*π*^	6	4.94E-02	-1.9	0.21	1.5	17	17	17
865	4	2.83E-02	-1.9	0.73	1.1	25	25	25
369	3	4.65E-02	-1.6			33	33	67
1604	4	2.57E-02	2.0	0.18	1.7	50	25	50
617	2	3.59E-02	2.2	0.85	-1.1	50	50	0
638	2	1.19E-02	2.4			50	50	50
747	2	4.13E-02	1.7	0.65	-1.1	50	50	0
1603	4	1.83E-02	3.2	0.02	3.2	50	50	50
2136	2	4.94E-02	-1.6	0.41	1.2	50	50	0
435	6	9.90E-03	-2.1	0.80	1.1	67	17	17
562	3	2.20E-03	7.5	0.97	1.0	67	33	33
1586	3	5.47E-03	3.1	0.39	-1.3	67	33	0
2535	3	2.38E-03	2.4	0.13	1.5	67	33	33
2213^*π*^	7	1.65E-02	-3.7	0.02	-3.7	71	29	0
1301	11	4.01E-02	2.7	0.86	-1.1	73	9	18
35	4	4.92E-02	1.9	0.80	-1.1	75	25	0
1014	4	3.48E-02	-1.7	0.48	1.3	75	25	0
619	4	2.28E-02	2.3	0.57	-1.1	75	75	0
867	13	1.05E-02	-2.2	0.20	1.4	77	15	8
1279	20	4.36E-02	1.6	0.59	-1.2	80	5	5
374	5	4.26E-02	2.3	0.64	-1.1	80	20	0
615	5	3.13E-03	5.1	0.68	1.1	80	60	20
119	6	1.77E-02	3.0	0.81	-1.1	83	17	0
1754	6	4.62E-02	1.7	0.88	1.1	83	17	17
866	9	1.45E-02	-1.9	0.84	1.1	89	22	0
1275	20	3.33E-02	-1.6	0.54	-1.1	100	10	0
2495	6	4.39E-02	2.9	1.00	1.0	100	17	0
858	4	3.87E-02	-1.8	0.18	-1.4	100	25	0
868	4	4.73E-02	-1.7	0.65	-1.2	100	25	0
336	3	2.46E-02	-1.8	0.52	-1.1	100	33	0
1090	3	4.84E-02	-1.6	0.11	-1.5	100	33	0
1508	6	7.64E-03	-2.0	0.50	-1.2	100	33	0
2178	3	3.87E-02	-1.8	0.72	-1.1	100	33	0
2384	3	3.69E-02	1.7	0.08	1.5	100	33	0
62	2	1.03E-03	2.8	0.31	1.3	100	50	0
441	2	6.63E-04	4.8	0.53	1.2	100	50	0
543	2	5.49E-03	3.5	0.50	1.2	100	50	0
1201	2	3.66E-03	2.2	0.11	1.4	100	50	0
1376	2	4.88E-02	46.2	0.24	1.6	100	50	0
2181	4	2.91E-02	-1.7	0.08	-1.5	100	50	0
2389	2	5.75E-03	2.	0.35	1.4	100	50	0
722	9	1.65E-02	-2.0	0.12	-1.5	100	67	0
1284	3	3.83E-02	-2.7	0.05	-2.8	100	67	0
1499	4	2.70E-02	2.4	0.07	1.8	100	75	0
1761	4	1.33E-03	4.2	0.63	1.1	100	75	0
550	4	7.51E-04	4.1	0.05	2.1	100	100	0
789	2	4.21E-02	-1.6	0.45	-1.5	100	100	0
881	2	2.67E-04	3.1	0.01	2.0	100	100	0
911	2	4.46E-02	-2.3	0.05	-1.9	100	100	0
1260	2	8.08E-06	8.6	0.00	14.3	100	100	0
1652	3	2.79E-05	14.8	0.01	2.6	100	100	0
1705	2	3.15E-02	-1.7	0.04	-1.6	100	100	0
1766	2	2.73E-03	2.7	0.01	2.6	100	100	0
2411	2	4.47E-03	2.4	0.01	1.8	100	100	0

*Table 3 contains a list of the genes in each of these operons.

^†^The lowest *p*-value from the CyberT analysis in each operon comparing the high concentration of MX (4.6 μM) to the control.

^‡^The fold change for the transcript with the lowest *p*-value in the operon.

^§ ^The *p*-value for the transcript with the highest *p*-value in the operon.

^¶ ^The fold change for the transcript with the highest *p*-value in the operon.

^¥^The percent of transcripts in the operon with the same direction of change as the transcript with the lowest *p*-value in the operon.

^#^The percent of genes where the intensity measurements were equal to or less than background.

^*π*^These operons had the majority of their genes expressing in the direction opposite that of a gene with a *p*-value < 0.05. Operon #2213 had two genes with *p*-values < 0.05 that changed in opposite directions.

**Table 3:** Genes in 54 operons identified by operon analysis

Operon number	Genes in operon
35	yaaY-ribF-ileS-lspA
62	polB-STM0098
119	stfC-stfD-stfE-stfF-stfG-STM0201
336	ylbA-allC-allD
369	fes-ybdZ-entF
374	entC-entE-entB-entA-ybdB
435	kdpE-kdpD-kdpC-kdpB-kdpA-STM0707
441	STM0717-STM0718
543	STM0893-STM0894
550	STM0900-STM0901-STM0902-STM0903
562	STM0925-STM0926-STM0927
615	STM1005-STM1006-STM1007-STM1008-STM1009
617	STM1011-STM1012
619	STM1014-STM1015-STM1016-STM1017
638	STM1049-STM1050
722	flgB-flgC-flgD-flgE-flgF-flgG-flgH-flgI-flgJ
747	pepT-STM1228
789	yeaK-yeaJ
858	orf48-orf32-orf245-orf408
865	ssaB-ssaC-ssaD-ssaE
866	sseA-sseB-sscA-sseC-sseD-sseE-sscB-sseF-sseG
867	ssaG-ssaH-ssaI-ssaJ-STM1410-ssaK-ssaL-ssaM-ssaV-ssaN-ssaO-ssaP-ssaQ
868	ssaR-ssaS-ssaT-ssaU
881	nemA-ydhM
911	ydgF-ydgE
1014	STM1633-STM1634-STM1635-STM1636
1090	STM1733-yciC-yciB
1123	STM1786-STM1787-STM1788-STM1789-STM1790-STM1791
1201	ruvB-ruvA
1260	umuC-umuD
1275	cobT-cobS-cobU-cbiP-cbiO-cboQ-cbiN-cbiM-cbiL-cbiK-cbiJ-cbiH-cbiG-cbiF-cbiT-cbiE-cbiD-cbiC-cibB-cbiA
1279	pudB-pduC-pduD-pduE-pduG-pduH-pduJ-pduK-pduL-pduM-pduN-pduO-pduP-pduQ-pduS-pduT-pduU-pduV-pduW-pduX
1284	phsC-phsB-phsA
1301	manC-wcaI-wcaH-wcaG-gmd-wcaF-wcaE-wcaD-wcaC-wcaB-wcaA
1376	STM2236-STM2237
1499	cysA-cysW-cysU-cysP
1508	eutM-eutD-eutT-eutQ-eutP-eutS
1586	STM2586-STM2587-STM2588
1603	STM2628-STM2629-STM2630-STM2631
1604	STM2632-STM2633-STM2634-STM2635
1652	STM2726-STM2727-STM2728
1705	proV-proW
1754	STM2913-STM2914-ygbM-ygbL-ygbK-ygbJ
1761	ygbE-cysC-cysN-cysD
1766	cysI-cysJ
2136	yhhK-STM3566
2178	yhjS-yhjT-yhjU
2181	dppF-dppD-dppC-dppB
2213	yiaM-yiaN-yiaO-lyxK-sgbH-sgbU-sgbE
2384	ubiE-yigP-aarF
2389	STM3980-STM3981
2411	STM4030-STM4031
2495	thiH-thiG-STM4161-thiF-thiE-thiC
2535	dinF-STM4239-yjbJ

### Monotonic analysis

A final list of differentially expressed genes was then constructed by augmenting the previous list with monotonically changing genes as described in the Methods section. Monotonically changing genes are those whose expression steadily increased or decreased with each increase in concentration of MX. The analysis of monotonically changing transcript expression relative to MX concentration added 153 more genes to the list. The *p*-value distribution for these monotonically changing genes was also heavily weighted toward low *p*-values (Table 1). However, the operons containing monotonically changing genes did not show enrichment for genes with low *p*-values as did the operons identified by the CyberT analysis.

**Figure 3:**
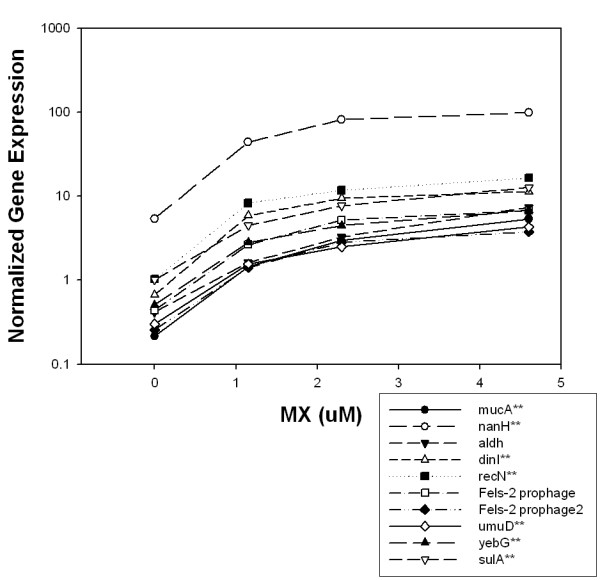
Monotonically increasing gene expression in MX-treated *Salmonella *TA100. The x-axis represents the concentration of MX at 4 concentrations, 0-, 1.15-, 2.3-, and 4.6-μM MX, respectively. The log-scaled y-axis represents expression values that are the mean of 12 background-corrected intensities (4 biological replicates and 3 technical replicates for each biological replicate) normalized to the DNA reference. Double asterisks represent those genes that are  known members of the LexA regulon.

**Figure 4:**
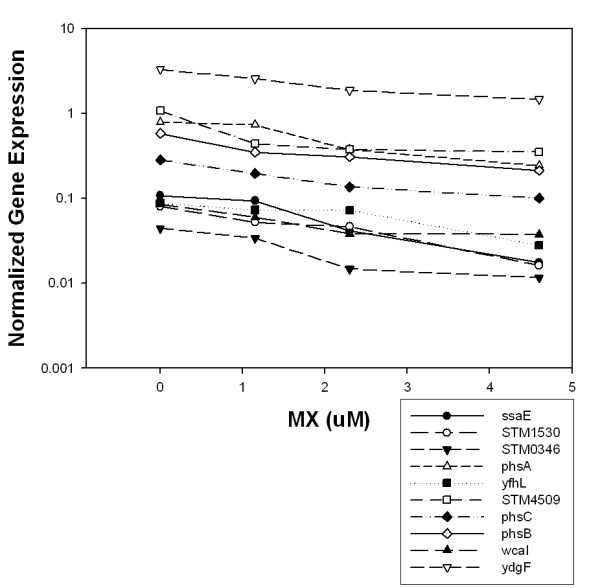
Monotonically decreasing gene expression in MX-treated *Salmonella *TA100. The x-axis represents the concentration of MX at 4 concentrations, 0-, 1.15-, 2.3-, and 4.6-μM MX, respectively. The log-scaled y-axis represents expression values that are the mean of 12 background-corrected intensities (4 biological replicates and 3 technical replicates for each biological replicate) normalized to the DNA reference.

For illustrative purposes, the 10 genes whose expressions were increased the most at the highest concentration of MX compared to the control and that showed a monotonic increase in expression are shown in Fig. 3. Seven of these 10 genes are involved in the SOS response. Fig. 4 similarly depicts the 10 genes whose expressions were monotonically decreased the most; *phsABC *is the thiosulfate reductase gene.

### LexA regulon analysis

The SOS response is the primary transcriptional response to DNA damage, and this response is mediated through the activation of the LexA regulon. The gene constituents of the LexA regulon have been studied intensely in *E. coli *[20,21]. Based on bioinformatic analysis, the gene constituents of the LexA regulon have been extended to *S. typhimurium *[22]. Table 4 contains the response of these LexA genes to the indicated concentrations of MX. The listed *p*-values were computed from the Cyber-T, Bayesian t-test, and the genes were ordered based on these *p*-values. Among those genes exhibiting significant expression (*p *< 0.05) in response to MX treatment (Table 4), most genes coded for proteins involved in DNA repair and replication [21]. Our finding that only 19 of the 33 genes of the *E. coli *LexA regulon were significantly expressed (*p *< 0.05) in *Salmonella *due to MX treatment suggests that the remaining 14 genes are not part of the LexA regulon of *Salmonella*.

**Table 4:** Fold change in expression of genes in LexA regulon due to MX treatment and normalized to control

		μM MX		
		
Gene	*p*-value*	0.25	0.5	1
		3.2	5.8	8.6
*STM2727*	0.00003	5.5	11.1	14.8
*dinP*	0.00003	3.7	4.8	4.8
*sulA*	0.00004	4.4	7.6	12.6
*umuD*	0.00004	5.2	8.3	14.3
*STM1019*	0.00005	4.8	8.4	8.9
*dinI*	0.00016	8.7	14	16.9
*yebG*	0.00019	5.5	8.8	13.5
*lexA*	0.00035	2.9	3.5	4
*STM1309*	0.00102	-1.3	1.9	2.5
*polB*	0.00103	2.3	3.1	2.8
*yigN*	0.00118	2.2	2.7	3
*uvrA*	0.00129	1.7	2.1	2.5
*recA*	0.00219	4	6.3	7.8
*uvrD*	0.00241	1.4	1.8	2.2
*ruvA*	0.00366	1.7	2.3	2.2
*sbmC*	0.00612	4.1	7.2	7.4
*ydjM*	0.00629	1.6	1.9	2.2
*recN*	0.02699	8	11.4	16
*yehR*	0.05804	1.1	0.8	9.6
*ftsK*	0.18374	1.3	1.2	1.4
*STM1056*	0.20943	0.9	1.3	1.5
*dinG*	0.4182	1	0.7	1.2
*uvrB*	0.43548	3.7	0.7	1.3
*minC*	0.46889	1	1	0.9
*mfd*	0.7057	0.9	1	0.9
*ftsY*	0.70953	1	1.1	1.1
*ybfE*	0.80643	0.1	0.9	0.9
*recG*	0.91485	1	0.9	1
*STM0925*	0.97301	1	0.9	1
*STM2621*	No Data			
*ssb*	No Data			
*yjiW*		1.8	1.4	1.9

*The *p*-value was determined by a Cyber-T t-test of control to the high concentration of MX.

### Functional analyses

Functional and pathway analyses were based on differentially expressed genes at all concentrations of MX determined from CyberT, operon analysis, and the analysis of monotonically changing gene expression. CyberT identified 169 altered genes, the operon analysis added 161 genes, and the monotonic analyses added 153 genes, for a total of 483 genes.

KEGG pathway analyses (Table 5) indicated that MX altered the expression of genes involved in cellular membrane and porphyrin metabolism, similar to the functions identified by the TIGR analyses (Table 6). Furthermore, the pathways and functions identified as altered (*p *< 0.05) after applying operon and monotonic analyses (483 genes) differed considerably from those identified by only CyberT analysis (169 genes) (Tables 5 and 6). In addition to the well-known effects of MX on DNA [6], our KEGG and TIGR analyses indicate that the pathway most altered by MX was porphyrin metabolism. In addition, MX also altered membrane function, specifically flagellar assembly and Type III secretion, two processes that have been shown recently to be co-regulated [23].

**Table 5:** Augmentation of differentially expressed genes in KEGG pathways by addition of operon and monotonic analyses to CyberT analysis

	CyberT		CyberT + operon + monotonic	
		
KEGG Pathway*	No. genes	*p*-value	No. genes	*p*-value
Porphyrin metabolism	2	0.230	19	4.75 × 10^-9^
Flagellar assembly	7	0.027	15	3.27 × 10^-5^
Type III secretion system	4	0.132	12	8.35 × 10^-5^
Sulfur metabolism	6	0.0002	7	0.0002
Thiamine metabolism	1	0.382	6	0.003
Nitrogen metabolism	0	NA	1	0.004
Biosynthesis of siderophore group peptides	1	0.338	4	0.005
Pyruvate metabolism	0	NA	1	0.008
Two-component system	0	NA	1	0.010
ABC transporters, prokaryotic	11	0.120	27	0.011
Selenoamino acid metabolism	5	0.010	6	0.014
Oxidative phosphorylation	0	NA	1	0.018
Other ion-coupled transporters	2	0.013	6	0.035
Ascorbate and aldarate metabolism	1	0.338	3	0.035
Arginine and proline metabolism	0	NA	1	0.037
HTH family transcriptional regulators	1	0.072	2	0.043

*Functions are ordered according to increasing *p*-value associated with operon and monotonic analyses.

**Table 6:** Augmentation of differentially expressed genes in TIGR functional groups by addition of operon and monotonic analyses to CyberT analysis

	CyberT		CyberT + operon + monotonic	
		
TIGR function*	No. genes	*p*-value	No. genes	*p*-value
Protein and peptide secretion and trafficking	6	0.013	21	1.86 × 10^-9^
Heme, porphyrin, and cobalamin biosynthesis	1	0.335	15	4.83 × 10^-7^
Thiamine biosynthesis	2	0.106	8	2.41 × 10^-5^
Transposon functions	14	0	16	0.0001
Regulatory functions	3	0.011	11	0.003
Unknown	41	0.003	115	0.010
Cell division; Prophage functions	2	0.002	2	0.011
Chemotaxis and motility	3	0.170	9	0.016
Anions transport and binding	4	0.013	6	0.018
Central intermediary metabolism	4	0.199	16	0.022
Biosynthesis of murein sacculus and peptidoglycan	1	0.266	1	0.033
DNA replication, recombination, and repair	13	0.0003	15	0.040
Sulfur metabolism	4	0.003	4	0.042
Amino acids and amines metabolism	0	NA	1	0.044
Surface structures	1	0.229	9	0.047
Transport and binding proteins	0	NA	1	0.048

*Functions are ordered according to increasing *p*-value associated with operon and monotonic analyses.

## Discussion

In an attempt to overcome the inherent noise from two-dye hybridization that obscures the statistical identification of differentially expressed genes, and to obtain a comprehensive list of genes whose expression changes were related to MX treatment, we applied two additional determinations of altered gene expression: an operon analysis and a monotonic analysis. Operon analysis increased the number of altered genes by a factor of 1.95. The Bayesian t-test *p*-value distribution for the genes that were added by operon analysis was heavily weighted towards low *p*-values as demonstrated by a hypergeometric test of this group (*p *< 1.7 × 10^-11^). Monotonic analysis increased the number of altered genes by a factor of 1.46. The *p*-value distribution for genes added by monotonic analysis was also heavily weighted toward low *p*-values (*p *< 1.0 × 10^-6^).

A total of 448 differentially expressed genes were subjected to functional analysis. Two broad categories for this functional response were those genes whose expression was altered in response to DNA damage and those whose expression was altered by other mechanisms. Among the former were genes involved directly in DNA damage repair and prophage excision. For those genes altered by other mechanisms, two predominant functions were (a) molecular transport and (b) porphyrin, heme, and cobalamin metabolism. Below we discuss these two functions as well as the possible role of *nanH *in the LexA regulon and concept of steric coupling to explain the down-regulation of porphyrin metabolism genes by MX.

### nanH and the LexA regulon

Bacteria partially regulate their response to DNA damage via the LexA regulon, which contains 33 genes in *E. coli *[21]. In *Salmonella*, we found that the expression of 19 of these genes was induced by MX (*p *< 0.05), and all showed a monotonic increase in expression with increasing MX concentration. One of the strongest responses to MX was exhibited by *nanH*, which codes for a neuraminidase/sialidase associated with pathogenicity in *Salmonella*. Although pathogenicity genes are not typically associated with the response to DNA damage, Benson et al. [22] identified a number of pathogenicity determinants induced as part of the SOS response. Because the *nanH *transcriptional response so closely matched the response of other genes in the LexA regulon, we investigated the potential for *nanH *membership in this regulon.

Modulation of the SOS response is facilitated by the differential affinity of LexA for the promotors of SOS response genes, which allows some genes to be fully induced at a lower level of DNA damage than others. The standard LexA binding site has a 16-bp palindromic repeat motif (CTGTN_8_ACAG) within 3–171 bp of the transcription site of the regulated gene [21]. For *nanH *there is a 21-bp palindromic sequence, CTGCTATATGTTATATAGCAG, where the middle three bp (underlined) do not participate in the palindrome. This potential regulatory sequence ends 20 bases prior to the *nanH *transcription start site, suggesting, along with the transcriptional evidence, that *nanH *may be regulated by LexA.

### Transport genes

Expression of a number of genes involved in transport processes was also affected by treatment with MX. Transporters serve numerous functions in bacteria, including uptake of nutrients, transport of proteins and peptides to the cell surface, transport of ions to regulate osmolarity, cell signaling, elimination of toxins, and secretion of virulence factors into host cells. Two well-studied prokaryotic transport systems are the Type III Secretion system and the ABC Transport system. In *Salmonella*, the Type III system seems to be involved mainly with host-cell interactions through secretion of virulence proteins, whereas the ABC Transport system supports a wide range of functions, including host-cell interactions and physiological maintenance of the bacteria cell itself. In this study, MX induced expression changes in genes involved in both the Type III secretion and the ABC Transport systems. In general, Type III Secretion system genes were down-regulated, whereas the ABC Transport system genes were either down- or up-regulated depending on their transport function. Thus, the expression of genes encoding proteins involved in amino acid transport was down-regulated, whereas that of genes encoding proteins involved in ion transport was up-regulated.

Not surprisingly, our results show that the potent mutagen MX activates DNA repair genes in *Salmonella*. In mammalian systems MX-induced DNA damage seems to be efficiently repaired [24,25]. However, considering the other genes whose expression MX alters, it is unlikely that mutagenesis alone accounts for the carcinogenicity of MX. Our findings that MX alters the transcription of genes involved in other cellular processes, particularly membrane transport functions, and the recent report that MX is a potent inhibitor of gap-junction intercellular communication in rat cells in vitro [26,27] support the contention that MX disrupts cellular pathways that are important for differentiation, growth, and apoptosis. Indeed, MX reduced the expression of connexin43 protein in rat cells in vitro [26]. Thus, besides directly mutating key genes, MX also may alter expression of key genes, such as observed here for membrane transport and metabolism. These combined effects could contribute to the carcinogenic mechanism of MX.

### Steric coupling: MX and pyrrole

**Figure 5:**
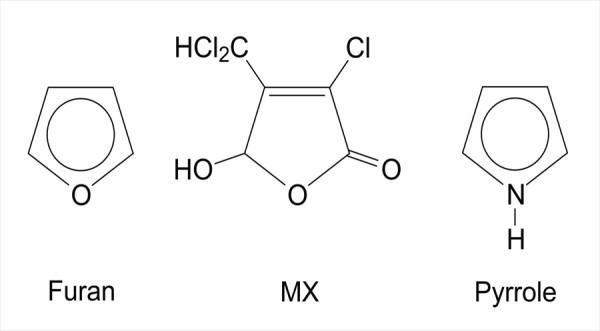
Structures of furan, MX, and pyrrole.

In order to explain the down-regulation of porphyrin metabolism by MX, we considered the possibility that a toxicant might disrupt a pathway and alter gene expression if it is structurally related, but not identical, to ligands in that pathway. MX contains a furan, which is a 5-membered ring containing an oxygen; this is structurally similar to a pyrrole, which contains a nitrogen instead of an oxygen in the ring (Fig. 5). Our data show that MX down-regulated genes involved in the metabolism of porphyrin, including its derivatives heme and cobalamin, both of which have pyrrole as a base structural unit. Heme is a component of red blood cells; however, in rats MX caused leukemia, which is a disease of the white blood cells [6]. MX also caused thyroid tumors in rats [6], and thyroxine contains two benzene rings connected by an oxygen atom, a structural element of MX.

These findings raise the interesting notion that some of the changes in gene expression resulting from toxicant treatment may be due to the structural similarity of the toxicant to effectors involved in normal cellular metabolism. The number of different small molecules within our own bodies may be just a few thousand [28], and these are processed by a select group of proteins to which they are linked sterically [29]. To our knowledge, consideration of the steric features of the toxicant has not been included in the interpretation of toxicogenomic data. However, our study suggests that such consideration may be beneficial to understanding why particular pathways are perturbed by a toxicant.

To distinguish between the possible modes of action of MX, we have initiated additional experiments with structural congeners of MX that have varying mutagenic potencies, different physical-chemical properties, and induce different mutation spectra–but are structurally similar to MX [30]. Such a study should indicate what components of the transcriptional response are due to which structural features of the mutagens and could segregate the DNA damage response from other transcriptional changes.

## Conclusion

The co-expression of similarly functioning genes has been demonstrated recently to be common not only to prokaryotes but also to eukaryotes from yeast to humans [31]. Thus, because transcriptional coupling exists in all species, the methodology described here is applicable to essentially all toxicogenomic assays, regardless of species. This methodology potentially identifies transcriptional impacts to cellular functions that might otherwise be overlooked. Combining transcriptional coupling in operons with the monotonic analysis can produce an analysis of toxicogenomic data that is more robust than that produced by CyberT analysis alone.

Such an analysis indicates that the drinking-water mutagen MX alters a variety of functions, including transporter activities and porphyrin metabolism. This latter effect of MX may be due to steric interference by MX, which is a furanone that bears structural similiarity to pyrrole. Our results also suggest that the *lexA *regulon of *Salmonella *may contain only 60% of the genes present in this regulon in *E. coli*. MX strongly induced expression of *nanH*, which contains a putative *lexA *regulatory motif in its regulatory region, suggesting that *nanH *may be a previously unrecognized member of the *lexA *regulon.

## Methods

### Cell growth, preparation, and treatment

The *S. typhimurium *base-substitution strain TA100 [*hisG46 chl-1005 (bio uvrB gal) rfa-1001 *pKM101^+^*Fels-1*^+^*Fels-2*^+^*Gifsy-1*^+^*Gifsy-2*^+^] [32] was grown in Oxoid Nutrient Broth No. 2 to log phase (2 × 10^8 ^cells/ml) and concentrated in PBS to ~10^10 ^cells/ml at 37°C. We added 2.9 ml of PBS, 1 ml of cell concentrate, and 100 μl of dimethyl sulfoxide (DMSO) or MX (gift from R. Franzen, Tampere University of Technology, Tampere, Finland) in DMSO to a 125-ml flask, which was then shaken at 120 rpm for 30 min at 37°C and then sampled for survival, mutagenesis, and RNA extraction. Cells were exposed to 1.15-, 2.3-, or 4.6-μM MX.

### Survival and mutagenesis

For survival, a sample was diluted 10^-6^, and 100 μl of this dilution were plated in triplicate onto VBME medium supplemented with excess biotin and histidine [33]. For mutagenesis, 50 μl of the treated cells were plated in duplicate onto VBME supplemented with excess biotin and trace histidine [33]. Plates were incubated for 3 days, and the colonies were counted by an automatic colony counter. Immediately after sampling for survival and mutagenesis, RNA was extracted and purified using the Institute of Food Research Microarray Facility protocol [34]. Mutagenesis and microarray results were the average of four independent experiments.

### Microarray procedures

Array fabrication, DNA and RNA labeling, and hybridizations for all biological samples were performed as described [16]. Briefly, we used glass slides arrayed in triplicate with PCR products from the genome of S *almonella *LT2 plus the genes from the pKM101 plasmid, i.e., each gene was spotted three times per slide, for a total of 4366 genes. RNA was isolated from treated and control cells and converted to cDNA, which was competitively hybridized with genomic DNA from control TA100 cells. For each treatment condition, two biological samples were hybridized reciprocally such that the genomic DNA was labeled with Cy3 and the cDNA with Cy5, and then vice versa. This was repeated for two more biological samples, yielding four competitive hybridizations per treatment condition. The slides were scanned on a GenePix 4000B Axon Scanner (Axon Instruments, Inc., Union City, CA), and signal intensities were quantified using the GenePix TM Pro 4.1 software package (Axon Instruments, Inc., Union City, CA). Consequently, the intensity data obtained for expression analysis for each gene were derived from 12 separate measurements (4 independent biological samples × 3 technical replicates per sample). These intensity data have been archived in the Gene Expression Omnibus [35], accession number GSE7034.

### Initial data analyses and concentration-related changes

Data were first analyzed as described previously [16]. Briefly, spot intensities were quantified, the local background intensities were subtracted, and the ratios of the contribution of each spot to total signal in each channel were calculated. Because each gene was spotted three times per slide, the average of the three replicate set of genes/slide constituted the measure of expression of a gene for each experiment.

### Montonic analysis

Montonically changing genes were those whose expression fulfilled the following conditions: (a) exhibited a progressive increase or decrease in expression across all 3 concentrations of MX and (b) changed by ≥ 50% between the control and the highest concentration of MX.

### Functional analysis

The functions of the differentially expressed genes identified above were ascribed to each gene using The Institute for Genomic Research (TIGR) Comprehensive Microbial Resource [36]. KEGG pathway annotations were obtained from Kyoto Encyclopedia for Genes and Genomes (KEGG) [37]. Pathway *p*-values for gene constituent over-representation were calculated by a hypergeometric test in Excel.

## Abbreviations

DMSO – dimethyl sulfoxide

DNA – deoxyribonucleic acid

KEGG – Kyoto Encyclopedia for Genes and Genomes (KEGG)

MX – 3-chloro-4-(dichloromethyl)-5-hydroxy-2(5*H*)-furanone

PBS – phosphate buffered saline

TIGR – The Institute for Genome Research

## Authors' contributions

WOW performed hybridization experiments, did all of the bioinformatic analyses, and helped write the manuscript. CDS performed hybridization and mutagenesis experiments and helped write the manuscript. NMH performed hybridization experiments. SHW performed mutagenesis experiments. GWK performed RT-PCR. SP and MM designed and made the microarray chips and aided in data analysis. DMD conceived of the study, participated in its design and coordination, and helped write the manuscript. All authors read and approved the final manuscript.

## References

[B1] Carpentier AS, Riva A, Tisseur P, Didier G, Henaut A (2004). The operons, a criterion to compare the reliability of transcriptome analysis tools: ICA is more reliable than ANOVA, PLS and PCA. Computational Biol Chem.

[B2] Harr B, Schlotterer C (2006). Comparison of algorithms for the analysis of Affymetrix microarray data as evaluated by co-expression of genes in known operons. Nucleic Acids Res.

[B3] Xiao G, Martinez-Vaz B, Pan W, Khodursky AB (2006). Operon information improves gene expression estimation for cDNA microarrays. BMC Genomics.

[B4] Price MN, Arkin AP, Alm EJ (2006). OpWise: operons aid the identification of differentially expressed genes in bacterial microarray experiments. BMC Bioinformatics.

[B5] Hu J, Kapoor M, Zhang W, Hamilton SR, Coombes KR (2005). Analysis of dose-response effects on gene expression data with comparison of two microarray platforms. Bioinformatics.

[B6] McDonald TA, Komulainen H (2005). Carcinogenicity of the chlorination disinfection by-product MX. J Environ Sci Health.

[B7] Woo YT, Lai D, McLain JL, Manibusan MK, Dellarco V (2002). Use of mechanism-based structure-activity relationships analysis in carcinogenic potential ranking for drinking water disinfection by-products. Environ Health Perspect.

[B8] Amundson SA, Do KT, Vinikoor L, Koch-Paiz CA, Bittner ML, Trent JM, Meltzer P, Fornace AJ (2005). Stress-specific signatures: expression profiling of p53 wild-type and -null human cells. Oncogene.

[B9] Seidel SD, Kan HL, Stott WT, Schisler MR, Gollapudi BB (2003). Identification of transcriptome profiles for the DNA-damaging agents bleomycin and hydrogen peroxide in L5178Y mouse lymphoma cells. Environ Mol Mutagen.

[B10] Muller A, Boitier E, Hu T, Carr GJ, Le Fevre AC, Marchandeau JP, Flor M, Jefferson F, Aardema MJ, Thybaud V (2005). Laboratory variability does not preclude identification of biological functions impacted by hydroxyurea. Environ Mol Mutagen.

[B11] Luo W, Fan W, Xie H, Jing L, Ricicki E, Vouros P, Zhao LP, Zarbl H (2005). Phenotypic anchoring of global gene expression profiles induced by N-hydroxy-4-acetylaminobiphenyl and benzo[a]pyrene diol epoxide reveals correlations between expression profiles and mechanism of toxicity. Chem Res Toxicol.

[B12] Ricicki EM, Luo W, Fan W, Zhao LP, Zarbl H, Vouros P (2006). Quantification of *N *-(deoxyguanosin-8-yl)-4-aminobiphenyl adducts in human lymphoblastoid TK6 cells dosed with N-hydroxy-4-acetylaminobiphenyl and their relationship to mutation, toxicity, and gene expression profiling. Anal Chem.

[B13] Guo Y, Breeden LL, Fan W, Zhao LP, Eaton DL, Zarbl H (2006). Analysis of cellular responses to aflatoxin B_1 _in yeast expressing human cytochrome P450 1A2 using cDNA microarrays. Mutat Res.

[B14] Jelinsky SA, Samson LD (1999). Global response of *Saccharomyces cerevisiae *to an alkylating agent. Proc Natl Acad Sci USA.

[B15] Jelinsky SA, Estep P, Church GM, Samson LD (2000). Regulatory networks revealed by transcriptional profiling of damaged *Saccharomyces cerevisiae *cells: Rpn4 links base excision repair with proteasomes. Mol Cell Biol.

[B16] Porwollik S, Frye J, Florea LD, Blackmer F, McClelland M (2003). A non-redundant microarray of genes for two related bacteria. Nucleic Acids Res.

[B17] Baldi P, Long AD (2001). A Bayesian framework for the analysis of microarray expression data: regularized t -test and statistical inferences of gene changes. Bioinformatics.

[B18] de Hoon MJ, Imoto S, Nolan J, Miyano S (2004). Open source clustering software. Bioinformatics.

[B19] Salgado H, Santos-Zavaleta A, Gama-Castro S, Millan-Zarate D, Diaz-Peredo E, Sanchez-Solano F, Perez-Rueda E, Bonavides-Martinez C, Collado-Vides J (2001). RegulonDB (version 3.2): transcriptional regulation and operon organization in *Escherichia coli *K-12. Nucleic Acids Res.

[B20] Fernandez De Henestrosa AR, Ogi T, Aoyagi S, Chafin D, Hayes JJ, Ohmori H, Woodgate R (2000). Identification of additional genes belonging to the LexA regulon in *Escherichia coli *. Mol Microbiol.

[B21] Erill I, Escribano M, Campoy S, Barbe J (2003). In silico analysis reveals substantial variability in the gene contents of the gamma proteobacteria LexA-regulon. Bioinformatics.

[B22] Benson NR, Wong RM, McClelland M (2000). Analysis of the SOS response in *Salmonella enterica *serovar typhimurium using RNA fingerprinting by arbitrarily primed PCR. J Bacteriol.

[B23] Soscia C, Hachani A, Bernadac A, Filloux A, Bleves S (2007). Cross-talk between type three secretion and flagellar assembly in *Pseudomonas aeruginosa *. J Bacteriol.

[B24] Marsteinstredet U, Brunborg G, Bjoras M, Soderlund E, Seeberg E, Kronberg L, Holme JA (1997). DNA damage induced by 3-chloro-4-(dichloromethyl)-5-hydroxy-2[*5H *]-furanone (MX) in HL-60 cells and purified DNA in vitro. Mutat Res.

[B25] Holme JA, Haddeland U, Haug K, Brunborg G (1999). DNA damage induced by the drinking water mutagen 3-chloro-4-(dichloromethyl)-5-hydroxy-2[*5H *]-furanone (MX) in mammalian cells in vitro and in mice. Mutat Res.

[B26] Hakulinen P, Rintala E, Maki-Paakkanen J, Komulainen H (2006). Altered expression of connexin43 in the inhibition of gap junctional intercellular communication by chlorohydroxyfuranones in WB-F344 rat liver epithelial cells. Toxicol Appl Pharmacol.

[B27] Nishikawa A, Sai K, Okazaki K, Son HY, Kanki K, Nakajima M, Kinae N, Nohmi T, Trosko JE, Inoue T (2006). MX, a by-product of water chlorination, lacks in vivo genotoxicity in *gpt *delta mice but inhibits gap junctional intercellular communication in rat WB cells. Environ Mol Mutagen.

[B28] Goto S, Okuno Y, Hattori M, Nishioka T, Kanehisa M (2002). LIGAND: database of chemical compounds and reactions in biological pathways. Nucleic Acids Res.

[B29] Dobson CM (2004). Chemical space and biology. Nature.

[B30] DeMarini DM, Landi S, Ohe T, Shaughnessy DT, Franzen R, Richard AM (2000). Mutation spectra in Salmonella of analogues of MX: implications of chemical structure for mutational mechanisms. Mutat Res.

[B31] Stuart JM, Segal E, Koller D, Kim SK (2003). A gene-coexpression network for global discovery of conserved genetic modules. Science.

[B32] Porwollik S, Wong RM, Sims SH, Schaaper RM, DeMarini DM, McClelland M (2001). The Δ*uvrB *mutations in the Ames strains of *Salmonella *span 15 to 119 genes. Mutat Res.

[B33] Maron DM, Ames BN (1983). Revised methods for the Salmonella mutagenicity test. Mutat Res.

[B34] Institute of Food Research Microarray FacilityProtocol. http://www.ifr.bbsrc.ac.uk/Safety/Microarrays/protocols.html.

[B35] Gene Expression Omnibus. http://www.ncbi.nlm.nih.gov/geo/.

[B36] The Institute for Genomic Research (TIGR) Comprehensive Microbial Resource. http://cmr.tigr.org/tigr-scripts/CMR/CmrHomePage.cgi.

[B37] Kyoto Encyclopedia for Genes and Genomes (KEGG). http://www.genome.jp/kegg/.

